# Melioidosis with Septic Shock and Disseminated Infection in a Neutropenic Patient Receiving Chemotherapy

**DOI:** 10.1155/2020/8976180

**Published:** 2020-03-25

**Authors:** Sitthi Sukauichai, Chantana Pattarowas

**Affiliations:** ^1^Chonburi Cancer Hospital, Department of Chemotherapy, Chonburi, Thailand; ^2^Chonburi Cancer Hospital, Department of Radiology, Chonburi, Thailand

## Abstract

Melioidosis is a bacterial infection, caused by Gram-negative bacillus, *Burkholderia pseudomallei*, widespread in Southeast Asia and the northern part of Australia, resulting in a high mortality rate in severe infection. However, it has rarely been reported in patients with chemotherapy-induced neutropenia. The authors described a case of melioidosis in a neutropenic patient presenting with septic shock after receiving chemotherapy. Blood and urine cultures were positive for *Burkholderia pseudomallei*, and CT scan showed multiple pulmonary nodules and hepatosplenic abscesses. The patient was successfully treated with antibiotics for the infection and with combined modalities for a malignancy.

## 1. Introduction

Melioidosis is a tropical bacterial infection originated from a Gram-negative bacillus, *Burkholderia pseudomallei*. The organism is found in soil and water in an endemic area, especially in Southeast Asia and the northern part of Australia [1]. Clinically, melioidosis presents in various manifestations based on duration of onset, severity of disease, or the number of organ involvements that are localized or disseminated infections, primary bacteremia, or even fatal septic shock [1, 2]. However, it is a relatively unusual pathogen in cancer patients [3]. The purpose of this article is to report a case of febrile neutropenia with septic shock, caused by melioidosis, and the case is complicated by locally advanced inoperable breast cancer.

## 2. Case Presentation

**Figure 1:**
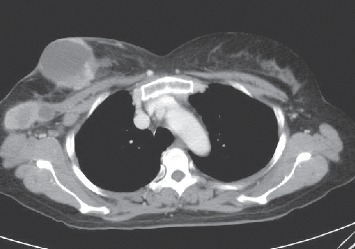
CT scan showed mixed solid-cystic mass in the right breast with matted necrotic right axillary node involvement.

A 54-year-old Thai female with locally advanced breast cancer (T4N2M0) stage III-B and hepatitis B carrier presented with a large right breast lump with skin involvement. She was a housewife, lived in Chonburi Province in the east of Thailand, and had no other medical conditions. The tumor's profile was grade 3 invasive ductal carcinoma, estrogen and progesterone receptors negative, and human epidermal receptor 2 positive. Physical exam showed right breast mass 10 × 10 cm with skin inflammation and right axillary lymph node 9 cm, and others were unremarkable. Computed tomography (CT) scan of the chest and abdomen was done for staging and showed no evidence of distant metastasis (Figure 1).

She received chemotherapy consisting of Adriamycin (doxorubicin) and cyclophosphamide (AC) as neoadjuvant treatment and lamivudine for hepatitis B infection. Unfortunately, she had two episodes of febrile neutropenia (FN) after the chemotherapy although she received granulocyte-colony stimulating factor (G-CSF) subcutaneously for FN prophylaxis in the second cycle of the chemotherapy. The both episodes were successfully treated with intravenous (IV) ceftazidime together with G-CSF, and blood cultures were negative. She received AC a little bit behind the schedule (normally every 3 week); however, her breast tumor was shrunk after the second cycle.

In the third cycle, she received decreased dose AC plus G-CSF prophylaxis. Nonetheless, on day 10 after the chemotherapy, she presented with fatigue, diarrhea, and fever. Initial physical examination was as follows: blood pressure 88/51 mmHg, pulse rate 124/min, body temperature 39.5°C, respiratory rate 32/min, and O_2_ saturation 88% on room air. She looked ill, marked icteric sclerae. Cardiovascular exam revealed regular rhythm, tachycardia, and no murmur. Other systems were unremarkable. Sepsis and febrile neutropenia were the most likely diagnosis.

After blood cultures were done, she received IV piperacillin/tazobactam as empirical antibiotic, IV fluid, and G-CSF injection and was admitted to a hospital. Laboratory tests showed CBC: hemoglobin 6.4 g/dL (10.8–14.2), white blood cell count 450 cells/*μ*L (3,700–10,000), absolute neutrophil count 230 cells/*μ*L (1,600–6,900), platelet 26,000 cells/*μ*L (150,000–360,000), Na 118 mmol/L (136–145), K 2.8 mmol/L (3.5–5.1), Cl 87 mmol/L (98–107), HCO_3_ 15 mmol/L (22–29), BUN 48 mg/dL (6–20), Cr 4.2 mg/dL (0.5–1.0), total bilirubin 4.7 mg/dL (0.0–1.2), direct bilirubin 4.6 mg/dL (0.0–0.2), albumin 3.2 g/dL (3.5–5.2), globulin 3.7 g/dL, AST 368 U/L (0–32), ALT 387 U/L (0–33), and ALP 154 U/L (35–104); urine: SpGr 1.025 (1.003–1.030), protein 2+, WBC 2-3 cells/HPF, and bacteria-numerous. On the same day, 8 hours later, she deteriorated and needed to receive orotracheal tube intubation, mechanical ventilator support, and inotropic agent; moreover, the antibiotic was changed to imipenem and metronidazole. The patient was diagnosed with septic shock with multiorgan failure, febrile neutropenia with underlying breast cancer, and hepatitis B infection.

**Figure 2:**
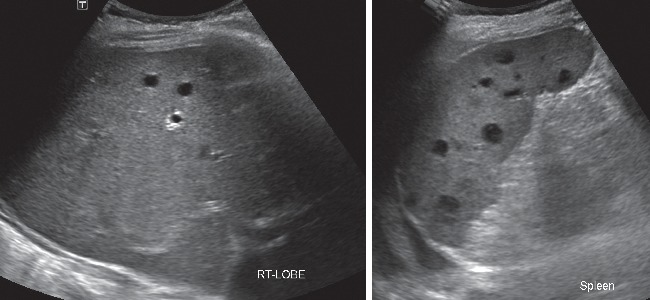
Ultrasound showed multiple hypoechoic nodules in the liver and spleen, compatible with abscesses.

**Figure 3:**
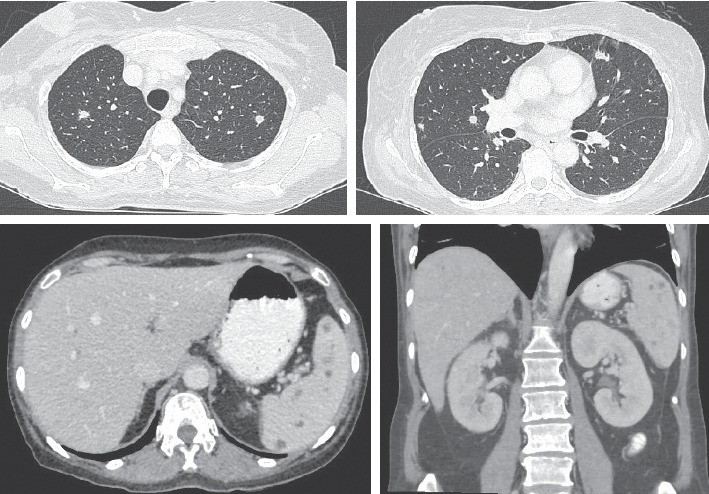
CT revealed multiple irregular small discrete nodules in both lungs size <1 cm and multiple nonenhanced hypoechoic nodules in the liver and spleen, microabscesses.

With intensive care, her symptoms were gradually improved. Dopamine and the orotracheal tube were off on day 4 and day 5 after admission, respectively. On day 6, two blood cultures and a urine culture showed positive for *Burkholderia pseudomallei* susceptible to amoxicillin-clavulanate, ceftazidime, imipenem, and trimetroprim/sulfamethoxazole (TMP/SMX), and stool exam for *Clostridium difficile* toxin revealed negative. Thus, the antibiotic was de-escalated to ceftazidime 2 gram IV every 8 hour, which was infused until 14 days. Fever disappeared on day 10 after injection of ceftazidime, and on the same day, ultrasound of upper abdomen showed multiple small hypoechoic nodules of the liver and spleen (Figure 2). A repeated blood culture was negative. On day 20 after admission, CT scan of the chest and whole abdomen was done, showing small lung nodules and multiple small hepatosplenic abscesses, which was compatible with disseminated melioidosis, and breast tumor was decreased in size (Figure 3). She was discharged on the same day and received TMP/SMX 3 tablets two times a day.

**Figure 4:**
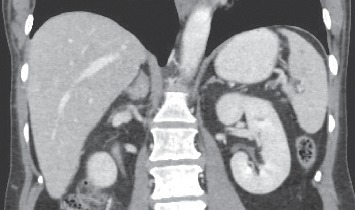
CT showed decreased multiple hypoechoic nodules in the liver and spleen.

At the 3rd month after treatment with TMP/SMX for melioidosis and changing chemotherapy combined with trastuzumab for breast cancer, she had no symptom from infection, but breast tumor had progressed in size. CT scan was repeated to evaluate diseases and showed the breast tumor increased in size, but markedly decreased in the number of lung, liver, and splenic nodules (Figure 4). As a consequence, she received TMP/SMX up to 20 weeks, and preoperative concurrent chemoradiation for breast tumor was performed. Finally, mastectomy was done successfully, and the pathological report showed 3.5 cm residual tumor, lymph node positive 11/12 for metastasis, and free surgical margin. CT scan was repeated again 3 months later, showing no evidence of recurrent tumor and infection.

## 3. Discussion

Melioidosis is an environmental bacterial infection, caused by *Burkholderia pseudomallei*. It can infect humans living in endemic areas via skin penetration, inhalation, and ingestion [1]. A variety of clinical manifestations can be found, ranging from chronic localized skin infection to severe pneumonia, or even to acute fulminant bacteremia [2]. Moreover, it can also disseminate to visceral organs causing abscesses, including hepatic, splenic, renal, or prostatic abscesses or causing bone, joint, and central nervous system infections [1, 2]. Most patients often have preexisting medical conditions or risk factors that include diabetes mellitus, chronic renal disease, chronic lung disease, thalassemia, alcohol use, and occupational contact to soil or water [2, 3].

Fever in patients with chemotherapy-induced neutropenia is a serious condition. Chemotherapy in breast cancer, AC regimen, results in FN in 2.5 to 25% of patients, depending on race and body mass index [4–7]. Although causative pathogens in FN with bacteremia tend to shift to Gram-positive bacteria in patients with indwelling catheter, Gram-negative bacilli remain the most common pathogens in neutropenic patients, especially *Escherichia coli*, *Klebsiella pneumoniae*, and *Pseudomonas aeruginosa* [8–10]. Nonetheless, *Burkholderia pseudomallei* is a rare pathogen responsible for a bacteremia in this setting. Since 1980, there have been a few reports of melioidosis with febrile neutropenia (Table 1) [11, 12]. In addition, all of these were from endemic areas.

Additionally, melioidosis is a unique bacterial infection in terms of resistance to penicillin, first- and second-generation cephalosporin, and aminoglycosides, high mortality rate up to 50% in patients with septic shock, and potentially relapsed infection [1, 2]. Treatment is composed of two phases: acute phase is a life-saving period using an intravenous ceftazidime, imipenem, or meropenem, while eradication phase is to prevent relapse with long-term treatment, up to 20 weeks, by an oral antibiotic: TMP/SMX or amoxicillin/clavulanic acid [13].

Fortunately, antibiotics for empirical treatment of FN and for acute phase of melioidosis are similar. This patient initially received piperacillin/tazobactam, but her symptoms got worse; thus, the antibiotic was changed to imipenem, in combination with intensive care. As a result, she gradually improved and survived. According to the recommendation, in the acute phase, this patient received ceftazidime for 14 days. In addition, she needed to receive an eradication phase antibiotic, TMP/SMX, to get rid of disseminated infection in her viscera and prevent being relapsed. At the same time, she was treated with the combination of chemotherapy, targeted therapy, and radiation for breast cancer, the purpose of which was to remove the tumor and cure from the malignancy.

Furthermore, the patient had liver and splenic abscesses which showed small and discrete nodules by imaging, and the abscesses of both organs occurred concurrently. These features were typical and highly suggestive for melioidosis rather than for other pyogenic pathogens [14]. Moreover, lung imaging in this patient showed generalized small discrete nodules compatible with blood-borne infection [15].

Lastly, melioidosis should be in the list of differential diagnosis in neutropenic patients with septic shock, especially who are living in the endemic area and having risk factors. Early diagnosis and prompt treatment, including proper antibiotics and intensive care, help the patient recover and survive.

## 4. Conclusion

The authors presented a case of melioidosis in a neutropenic patient receiving chemotherapy. Although she suffered from septic shock, disseminated melioidosis, and locally advanced breast cancer, eventually she made a recovery from infection and underwent mastectomy for the tumor.

## Figures and Tables

**Table 1:** Melioidosis with febrile neutropenia during receiving chemotherapy.

Country (year)	Sex-age	Underlying condition	(A) Cancer	Initial WBC/ANC	Empirical antibiotics	Site of (A) infection	Outcome
			(B) chemotherapy received			(B) culture+	
Malaysia (1980)	M-13	None	(A) ALL	1100/825	Cloxacillin + kanamycin	(A) cellulitis + septicemia	Died, day 6
			(B) VAMP			(B) blood	
India (2010)	F-46	Hypothyroid	(A) Ovary	1500/NA	Teicoplanin + cefipime	(A) pneumonia + septicemia	Died, rapidly
			(B) not reported			(B) blood	
	M-35	None	(A) Stomach	NA/NA	Cefipime	(A) pneumonia	Survived
			(B) 5FU/LV + RT			(B) sputum and BAL	
Thailand (2020) present case	F-54	Hepatitis B carrier	(A) Breast	450/230	Pip/Tazo then imipenem	(A) septicemia, liver, and spleen abscesses	Survived
			(B) AC			(B) blood and urine	

AC = Adriamycin/cyclophosphamide; ANC = absolute neutrophil count (cells/*μ*L); ALL = acute lymphoblastic leukemia; BAL = bronchoalveolar lavage; F = female; M = male; NA = not available; Pip/Tazo = piperacillin/tazobactam; VAMP = vincristine/Adriamycin/6-mercaptopurine/prednisolone; WBC = white blood cell count (cells/*μ*L).
